# Heparin-Binding Growth-Associated Molecule (Pleiotrophin) Affects Sensory Signaling and Selected Motor Functions in Mouse Model of Anatomically Incomplete Cervical Spinal Cord Injury

**DOI:** 10.3389/fneur.2021.738800

**Published:** 2021-12-06

**Authors:** Natalia Kulesskaya, Dmitry Molotkov, Sonny Sliepen, Ekaterina Mugantseva, Arturo Garcia Horsman, Mikhail Paveliev, Heikki Rauvala

**Affiliations:** ^1^Neuroscience Center, Helsinki Institute of Life Science, University of Helsinki, Helsinki, Finland; ^2^Real-time Imaging Laboratory, Faculty of Pharmacy, University of Helsinki, Helsinki, Finland

**Keywords:** HB-GAM, pleiotrophin, spinal cord injury, animal models, BOLD signal

## Abstract

Heparin-binding growth-associated molecule (pleiotrophin) is a neurite outgrowth-promoting secretory protein that lines developing fiber tracts in juvenile CNS (central nervous system). Previously, we have shown that heparin-binding growth-associated molecule (HB-GAM) reverses the CSPG (chondroitin sulfate proteoglycan) inhibition on neurite outgrowth in the culture medium of primary CNS neurons and enhances axon growth through the injured spinal cord in mice demonstrated by two-photon imaging. In this study, we have started studies on the possible role of HB-GAM in enhancing functional recovery after incomplete spinal cord injury (SCI) using cervical lateral hemisection and hemicontusion mouse models. *In vivo* imaging of blood-oxygen-level-dependent (BOLD) signals associated with functional activity in the somatosensory cortex was used to assess the sensory functions during vibrotactile hind paw stimulation. The signal displays an exaggerated response in animals with lateral hemisection that recovers to the level seen in the sham-operated mice by injection of HB-GAM to the trauma site. The effect of HB-GAM treatment on sensory-motor functions was assessed by performance in demanding behavioral tests requiring integration of afferent and efferent signaling with central coordination. Administration of HB-GAM either by direct injection into the trauma site or by intrathecal injection improves the climbing abilities in animals with cervical hemisection and in addition enhances the grip strength in animals with lateral hemicontusion without affecting the spontaneous locomotor activity. Recovery of sensory signaling in the sensorimotor cortex by HB-GAM to the level of sham-operated mice may contribute to the improvement of skilled locomotion requiring integration of spatiotemporal signals in the somatosensory cortex.

## Introduction

The adult mammalian central nervous system displays a low capability to regenerate its connections after trauma. This has led to a plethora of experimental studies to enhance the regenerative potential in the central nervous system (CNS). A prominent reason for the regeneration failure of the CNS traumas such as spinal cord injury (SCI) is the hostile environment preventing the regrowth of neuronal connections (1, 2). Until now, an unmet medical need exists to develop therapies to enhance regeneration of neuronal connections after SCI and other CNS injuries.

In mature CNS, several types of chondroitin sulfate proteoglycans (CSPGs) with overlapping acute to chronic expression patterns provide an inhibitory environment for axon regeneration within several months after SCI (3). CSPGs accumulated in the glial scar after CNS injury are suggested to be major players in the regeneration failure in SCI and other CNS injuries (1). Importantly, the gradient of proteoglycans that exists in the glial scar with the highest CSPG concentration in the epicenter and the lowest in penumbra is ultimately required for axonal retraction and formation of dystrophic axonal endings (4, 5). In addition, CSPGs affect secondary injury and regeneration by inhibiting the oligodendrocyte precursor cells differentiation and migration (6) and by promoting pro-inflammatory phenotype in the microglia (7).

The concept of CSPG inhibition and its role in the regulation of CNS plasticity and regeneration has been largely based on the use of chondroitinase ABC that cleaves chondroitin sulfate chains of CSPGs or on inhibition of CSPG synthesis (1, 8–15). Indeed, multiple studies have demonstrated the beneficial effect of chondroitinase ABC treatment on axon regeneration and functional recovery after SCI (8, 16), although the core protein and the sugar stab remaining after the digestion could still provide some inhibitory activity (17–19). Recently, leukocyte common antigen-related (LAR) and protein tyrosine phosphatase sigma (PTPσ) were identified as CSPG receptors mediating the inhibitory effect on neurite outgrowth. Blocking of PTPσ or LAR signaling has been shown to enhance recovery from SCI in animal models (20, 21).

Heparin-binding growth-associated molecule (HB-GAM; pleiotrophin) was initially isolated as a neurite outgrowth-promoting protein that binds strongly to heparin (22). In addition to heparin, HB-GAM was found to bind avidly to CSPGs having similar sulfated carbohydrate epitopes as heparin (23). In solid-phase binding assays, HB-GAM inhibits PTPσ binding to chondroitin sulfate chains of the aggrecan matrix (24). HB-GAM is highly expressed in the early postnatal rodent brain corresponding to the critical period of development with heightened plasticity but is strongly downregulated upon adulthood (25). Furthermore, HB-GAM mRNA and protein expression is enhanced after CNS injuries (26, 27), suggesting participation in intrinsic repair mechanisms in the brain and spinal cord. Molecular cloning and studies on the expression profile of the HB-GAM protein and mRNA (25, 28) confirmed that HB-GAM is a novel secretory protein with properties that suggest a role in the development/plasticity of the CNS.

Our hypothesis proposes that HB-GAM could be an endogenously expressed regulator of plasticity and regeneration. This inference is based on avid binding to CSPGs (23), the neurite outgrowth-promoting property (22) and the prominent expression peak in the postnatal brain corresponding to the developmental period with high plasticity (25). Our recent studies have shown that HB-GAM may indeed be a regulator of CNS plasticity and regeneration since it is capable of reversing the neurite outgrowth inhibition by CSPGs such as aggrecan when tested with primary CNS neurons *in vitro* (24). Furthermore, two-photon imaging studies have shown that recombinant HB-GAM injected into the trauma site enhances axon growth through the injured spinal cord after thoracic dorso-lateral transection (24). However, functional consequences of HB-GAM administration in the recovery of sensory and motor functions after SCI have not been explored. To evaluate the functional regeneration after HB-GAM treatment we have elaborated in the current study a unilateral model of SCI that affects almost all ascending and descending tracts on one half of the body providing a broad range of symptomatic manifestations of sensory, motor, and sensory-motor modalities with advantages of behavioral asymmetry for internal control.

Most human SCIs occur at the cervical level (29) where partial injuries have a high incidence (30) while most pre-clinical studies in animals utilize the SCI model done at the thoracic level displaying some differences compared to the SCI at the cervical level (31). Therefore, we decided to focus our current work on SCI at the cervical level. Moreover, lateral hemitrauma at the cervical level results in prominent dysfunctions of the ipsilateral front paw and paw use asymmetry that can be assessed by multiple functional tests in laboratory animals in contrast to the SCI at the thoracic level where the choice of assessment tools for paw functions is much more limited (31, 32).

Lateral spinal cord hemisection is used as an animal model of the Brown-Séquard syndrome. Motor dysfunction and loss of light tactile sensations and proprioreception on the ipsilateral side of the trauma and loss of pain and temperature sensations contralaterally are the clinical symptoms of the Brown-Séquard syndrome. The symptom description in animal models is much more controversial ranging from loss of sensory functions to hyperalgesia and allodynia development (33–35). Moreover, some animals demonstrate the contralateral fingers and limbs autotomy (36) that reflects the development of neuropathic pain and results in severe local inflammation that would compromise the results of all further behavioral tests. Additionally, most behavioral tests of sensory functions depend a lot on the motivation, arousal, and emotional level of the animals that may alternate due to trauma causing inflammation and uncontrollable neuropathic pain. Therefore, we used *in vivo* brain imaging methods to reveal plastic alterations in the central representation of sensory functions to avoid the effects of motor and emotional peculiarities of the test animals on experimental results. Intrinsic signal optical imaging (ISOI) of the somatosensory cortex during hind paw vibrotactile stimulation was used to monitor the effect of HB-GAM local administration on sensory functions after lateral spinal cord hemisection at the cervical level in mice. An additional cohort of animals was used to study the effect of HB-GAM treatment on the recovery of motor and sensory-motor functions in the same SCI model.

To find a route for protein administration that is better compatible with human therapy and allows easily repeated dosing of the protein, we assessed the distribution of radioactively labeled HB-GAM to the thoracic and cervical areas after a single L5-L6 intrathecal injection. Repeated intrathecal administration of HB-GAM was tested in the mouse models of C5 hemisection and hemicontusion. Local injection to the trauma site and intrathecal injections into the cerebrospinal fluid give similar results in behavioral assays. Our studies suggest that HB-GAM induces recovery of sensory signaling in the sensorimotor cortex and promotes partial recovery of motor functions after SCI and might be valuable for human therapy.

## Materials and Methods

Adult (8–10 weeks at the beginning of the study) female C57BL/6NHsd and C57BL/6JRcc mice were used for functional and imaging studies respectively. Mice of C57BL6 strain are prone to develop ulcerative dermatitis that in severe cases could significantly impact animal behavior and general welfare (37). We found that in our studies, C57Bl6/NHsd mice develop dermatitis less frequently than C57Bl6/J, while it is not clear whether it depends on the genetic difference in C57Bl6 substrain or other non-genetical reasons. From the other side, our experience and published literature (38) suggested that C57Bl6/J could be more robust in multiple anesthesia exposures required for optical window installation and imaging sessions in blood-oxygen-level dependent (BOLD) studies, while C57BlL/6N animals demonstrated more mortality cases due to breathing dysfunctions and cardiac depression.

The mice were housed in groups of four to five animals in individually ventilated cages before surgery. Animals used for BOLD signal imaging were housed individually for a few days after optical window installation to secure tight frame fixation. Ventilated cages were set at a stable temperature (21°C) in a 12-h light/dark cycle and provided with food and water *ad libitum*.

### Hemisection and Hemicontusion Injuries

Before surgery, all animals were equally distributed based on the baseline vertical screen performance and body weight to the sham and the SCI groups. Animals received an antibiotic shot 30 min prior to the surgery and 3 days after the surgery (Borgal, 30 mg/kg, every 12 h). Painkillers were administered 15 min before the surgery and after the surgery during 2–3 days (Temgesic 0.1 mg/kg, every 12 h) depending on the animal recovery. In the first studies (BOLD imaging and motor performance studies in the hemisection model with local HB-GAM administration) we chose an injectable anesthesia cocktail (Ketamine + Rompun 8 ml/kg) to induce the trauma in order to avoid the use of inhalational masks and get better access to surgery area and more freedom to manipulate with an animal in technically challenging surgery followed by compound administration with microinjector. Inhaled isoflurane anesthesia (4%) was used for later functional studies (intrathecally administered HB-GAM in the hemisection and hemicontusion models), which improved animal survival, especially in the cervical hemicontusion model. Ketamine/xylazine injectable anesthesia results in cardiac (38) and respiratory depression (39), while isoflurane has a minor cardiac effects and provides stable steady-state concentration. For the surgery, animals were placed in the prone position on a heating plate (36°C) for body temperature maintenance. A tube (2 cm in diameter) was placed below the neck to lift the cervical region for better access. After skin shaving and disinfecting, skin and muscle incision was done on the cervical level to reveal vertebrae. T2 lamina with the prominent bone process was used as the landmark to identify the target area. The C5 bone lamina was removed to expose the spinal cord. To perform the hemisection, a 23 gauge needle was inserted vertically to the spinal cord right near the midline dorsal vessel and drugged to the lateral side. Bleeding was stopped by the application of small pieces of sterile hemostatic foam. Alternatively, hemicontusion trauma was done on the C5 spinal cord with the use of the contusion device Infinite Horizon Impactor (Precision Systems & Instrumentation, Lexington, KY, USA). Before the laminectomy procedure, the pair of custom-made metal spinal stabilizer bars (5 mm width) compatible with a standard mouse/neonatal rat adapter for the stereotaxic frame was positioned below the C5 vertebra transverse processes. After laminectomy, the animal was placed on the impactor device to position the C5 under the impactor tip (diameter 0.75 mm). The 75 kilodynes force impact with a dwell time of 15 s was produced to the right side of the spinal cord between the midline dorsal vessel and the lateral edge. Sham animals were subjected to similar surgical procedures including laminectomy but without an impact or needle cut application. After closing the muscles layers and the skin the animals were injected with 0.9% sodium chloride (NaCl) solution to prevent dehydration and placed in the warm recovery chamber for a few hours. Soft wet food and drinking bottles with long tips were provided for the next few days of the recovery period. Animal welfare, body weight, and efficiency of bladder emptying were monitored daily for 1 week after the trauma. Animals that were not recovered enough to be able to move and eat independently within 4 days after the surgery were excluded from the study.

### Local Spinal Cord Injections

Single injections of the recombinant HB-GAM characterized previously (40) (1 mg/ml) or vehicle (1x PBS) were done directly to the spinal cord lesion immediately after the spinal cord hemisection. Microinjector (UltraMicroPump with SYS Micro4 Controler, World Precision Instruments, Inc., Sarasota, Florida) with Hamilton syringe and attached glass electrode was used to administer 7 μl of solution slowly within 4 min.

### Intrathecal Injections

The mouse was anesthetized with isoflurane (2%) and placed in the prone position. The skin was shaved and disinfected in the lumbar area. The injection was done by a 31 G needle connected to Hamilton 10 μl syringe by polyethylene tubing. The L5 vertebra was localized between the iliac crests of the hip bones, and the needle was inserted through the skin between the L5 and L6 vertebras. After positioning of the needle in the intrathecal space verified by tail-flick reflex, 7 μl of solution (HB-GAM 1 mg/ml or 1xPBS) was slowly injected. The needle was kept at the place for an additional 15 s to prevent solution backflow. Intrathecal injections were repeated for 4 weeks once per week starting from day 7 after the spinal cord trauma. Sham animals were injected with PBS.

### SPECT-CT *in vivo* Imaging of HB-GAM Distribution

The HB-GAM protein was labeled with iodine-123 and purified from a free label at the Laboratory of Radiochemistry, University of Helsinki, using solid-state iodination of a single tyrosine in the protein sequence followed by high performance liquid chromatography (HPLC purification. The mice were anesthetized with isoflurane (4%); 7 μl of [I^123^]HB-GAM (35 μg of protein, 1.3 MBq) were injected through the skin by an intrathecal puncture to the lumbar area between the L5 and L6 vertebrae. Radioactivity in the syringe was measured prior to and post-injection. Single-photon emission computerized tomography-CT (SPECT-CT) imaging was carried out with NanoSPECT/CT (MEDISO, Hungary), a four-headed small animal scanner featuring a 1 mm multi-pinhole mouse apertures (AP3 6X hole, MEDISO, Hungary). SPECT images were acquired dynamically at around 30, 60, 90, and 120 min post-injection taking 24 projections at 90 s for two animals. Additionally, one mouse was imaged at 5 h and another at 15 h post-injection increasing the exposure to 120 s. CT imaging was acquired at 45 kVp in 180 projections, 500 mc/projection. SPECT images were reconstructed with HiSPECT NG software (Scivis GmbH, Göttingen, Germany) and fused with CT datasets with VivoQuant software (InVicro, Inc., USA). To quantify tracer uptake in spinal cord areas, reconstructed SPECT images were reoriented and analyzed with VivoQuant (InVicro, Inc., USA) software by using CT data as a reference. Three-dimensional (3D) volumes of interest (VOIs) were defined for three main spinal cord regions (cervical, thoracic and lumbar) by using the 3D ROI tool. The radioactivity in each VOI was calculated and results were presented as standard uptake value (SUV) calculated as the ratio of the activity in the VOI divided by the total activity injected per gram of mouse.

### Optical Window Installation and BOLD Imaging

The transparent skull optical window was installed as described previously (41). Briefly, animals were anesthetized with isoflurane and heads were fixed for the scalp and underlying tissue was removed. An equal cyanoacrylate priming layer (Loctite 401Glue, Henkel Adhesives, Germany) was applied to skull bone, followed by an acryl level applied twice with 30-min interval (Mixture acryl polymer powder, EuBeCos, Germany, with acryl liquid, Dentsply, USA). The next day the animals were anesthetized again, and the skull was polished using a fine-grit drill bit (Shofu fine grit size, PN0425 PC2) to reach the transparency of the skull. A metal round-shape frame with processes for fixation to imaging camera (RMB-9, Neurotar, Finland) was fixed by transparent nail polish layer to the transparent skull resulting in an optical window.

Intrinsic signal optical imaging (ISOI) was used to monitor local blood oxygenation changes associated with functional activity in the cortex. Animals were slightly anesthetized with isoflurane (0.9–1.2%), placed on a heating plate (36°C), and heads were fixed by implanted metal frames. Through the imaging experiment, the anesthesia level was monitored with a PhysioSuite physiological monitor (Kent Scientific, USA) and adjusted to keep the breath rate at around 120+/−10 breaths per minute. Custom vibrators (1 cm diameter) were fixed to the plantar surface of both hind paws for tactile stimulation. To obtain images of light reflectance, a 12-bit MV1-D1312-160 CL CMOS-camera (Photon Focus, Switzerland) equipped with paired objective lenses (Nikkor 135mm f/2.0 and inverted Nikkor 50mm f/1.2, Nikon Corporation, Japan) was used resulting in a 4 mm x 4 mm field of view. Image capture was done *via* Brain Imager 3001 imaging system laboratory interface (Optical Imaging Inc., Israel) with binning factor 4 and pixel resolution 12 x 12 microns. A drop of glycerol was then placed on the window and covered with a round 12 mm plastic coverslip to improve transparency. The camera was positioned according to a cortical functional map using the Bregma point as the skull landmark and cortical vessels to cover primary sensory regions for the hindlimbs. Prior to every imaging session, a map of cortical vasculature was captured with Green light illumination (Halogen light source with 546BP30 filters, Optical Imaging Inc., Israel). To image a stimulus-evoked BOLD signal, illumination of the cortex was done with red light [Halogen light source, 630BP30 filter (Optical Imaging Inc., Israel)], and an additional emission filter 590LP filter (Optical Imaging Inc., Israel) was placed in front of the camera to reduce possible short-wavelength noise. During the imaging session data, frames were captured with the acquisition frequency of 10 Hz and combined into 10 block files (300 data frames/block) resulting in 3,000 data frames per 5 min session. The sensory stimulation pattern (0.1 Hz on-off period) was synchronized with camera frame capturing *via* a custom MatLab script (MathWorks Inc., USA) and MCC USB-1209FS (Measurement Computing, USA) controller and resulted in a three-phased stimulus-evoked hemodynamic signal (42). Raw data block files were converted into 16-bit TIFF stacks in MatLab. Files were filtered with Gaussian blur (sigma; 7), to remove high-frequency spatial noise and the signal was masked out with Max Entropy threshold in ImageJ (FIJI software, open source plugin available at https://imagej.net/plugins/maximum-entropy-threshold). The outcome illustrates a 2D representation of the initial dip phase to perform surface area calculation. The magnitude of the initial dip phase was analyzed using Open-source FIJI software (available at https://imagej.net/software/fiji/).

### Behavioral Assays

#### Vertical Screen

The mouse was placed on the edge of the horizontal wire screen (25 x 22 cm, the diameter of wires 2 mm spaced at 1 cm) faced to the edge and then immediately turned with the screen vertically to the upside-down position. The latency to climb to the upper edge was measured with a 1 min cutoff time. The test was repeated in three subsequent trials with at least 1 min interval and the averaged value was used for data analysis. To understand better the role of ipsilateral front paw motor functions in vertical screen test performance we evaluated the usage of front paws in wire grasping before each testing session to detect possible paralysis or spasticity of paw/digits: the mouse was lifted by the tail and slowly move down to the horizontal wire screen allowing to extend the front paws and grasp the wire. The grasping abilities were scored between 0 and 2, where 0 is the normal functioning of ipsilateral paw, 1 is weak grasping with occasional paw displacement but relaxed paw and extended fingers after the wire release, and 2 is keeping paw flexed unable to grasp the wire.

#### Cylinder Test

The mouse was placed into the glass cylinder (diameter 13 cm, high 15 cm) and videotaped for the following analysis for the first 20 exploratory vertical rearings with 5 min cutoff time. Trials with <5 rearings were excluded from the analysis. An experimenter blinded to the animal group quantified the number of ipsilateral front paw use (ipsilateral alone plus both paws) and the total number of rearings with front paws used for bodyweight support against the wall.

#### Grip Strength Test

In the hemisection model, the grip strength was measured separately for ipsilateral and contralateral front paws keeping animals from the scruff and allowing them to grasp the bar with one paw only. Waiting until complete wound healing after the trauma surgery was required to start the grip strength testing (4 weeks). To be able to measure grip strength at an earlier time point (2 weeks) in the hemicontusion model, cumulative grip strength for both front paws was measured by an automated grip-strength meter (Ugo Basil, Italy). Animals handled by tail were allowed to grasp the horizontal metal bar. Mice were then gently pulled horizontally away from the bar until the grasp was released. Grip strength was measured as the maximum produced force averaged across five trials performed with at least 1 min intertrial interval.

#### Spontaneous Locomotor Activity in the Open Field

The animal was released to the corner of the open field chamber (30 cm × 30 cm, MedAssiociates, St. Albans, VT; illumination about 100lx) faced to the wall and allowed to explore the chamber for 15 min. Spontaneous locomotor activity was measured automatically by infrared sensors and analyzed by the Activity Monitor v5.1 software (Med Associates, St. Albans, VT) as the traveled distance and the rearing time (time spent standing on rear paws).

#### Von Frey Test

Mice were placed in individual restraining chambers (10 x 10 cm) with the wire-mesh floor for adaptation at least 30 min before the test. An electronic aesthesiometer (Ugo Basil, Italy) was used for the assessment of mechanical sensitivity and allodynia. A metal probe was placed under the central area of the hind paw palm, and pressure (constantly increasing from 0 to 5 g in 0.05 g steps; max 5 g) was applied until paw withdrawal. Latency to withdraw was analyzed for each hind paw as an average of four trials repeated with at least 2 min intertrial interval.

### Statistical Analysis

Statistical analysis was performed using GraphPad Prism 9, San Diego, CA. For normally distributed data two-way ANOVA followed by pairwise comparison with Tukey or Dunnett test was used for assessing the group difference at individual timepoints or within-group difference (between individual time points vs. 1 week) respectively. Non-normally distributed data were analyzed by Friedman test with Dunn's correction to compare individual time points (vs. 1 week) within the same group and Kruskal-Wallis test with uncorrected Dunn's pairwise comparison to test the group difference at individual time points. Data in the figures are presented as the mean +/– SEM.

## Results

### Local Injection of HB-GAM to the Trauma Site

**Figure 1:**
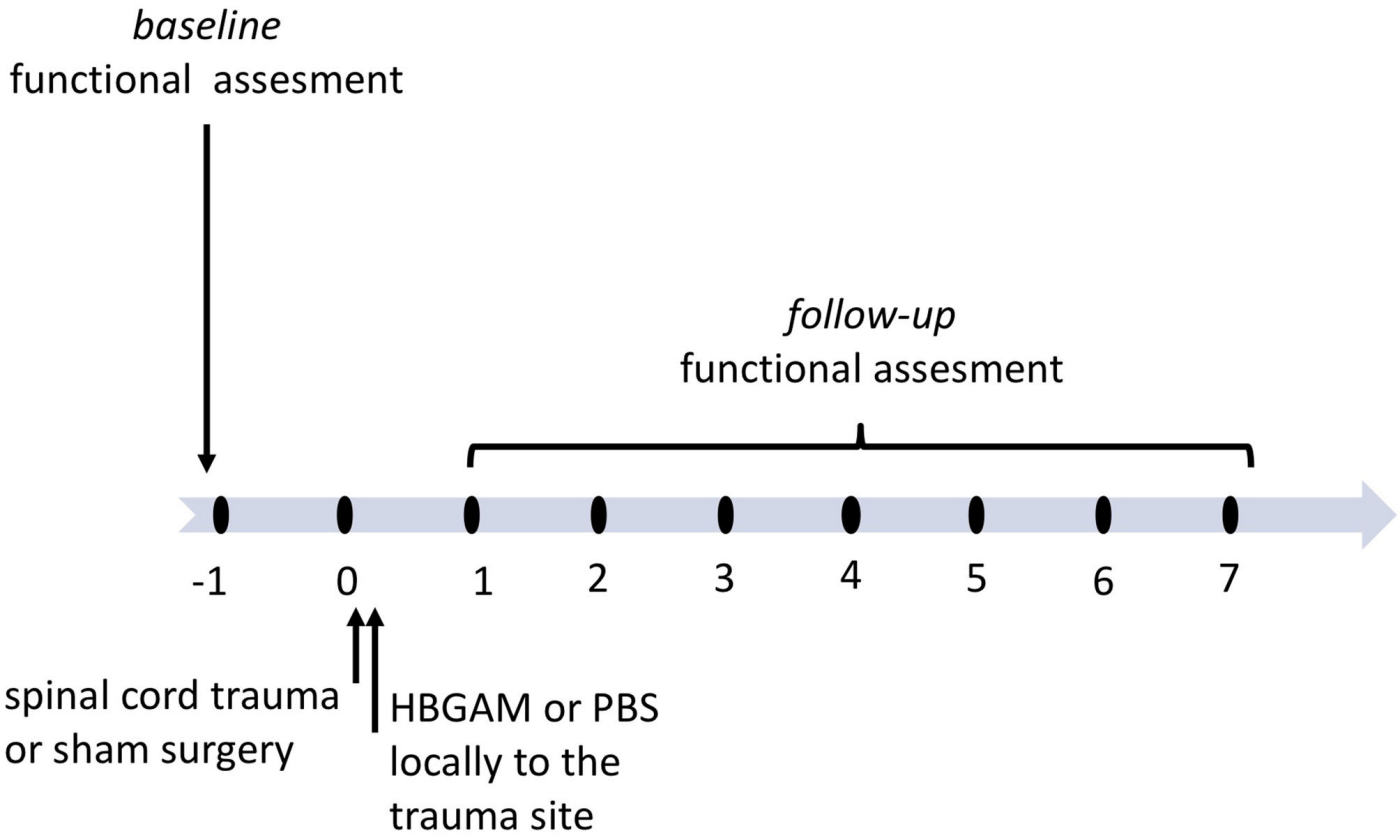
The schedule of treatment and functional assessment in the studies with locally administered HB-GAM or PBS in the C5 hemisection of the spinal cord. Animals with spinal cord trauma were equally distributed to the treatment groups or the sham group based on their baseline behavioral results or baseline BOLD imaging data measured one week before surgery. HB-GAM (7 μl, 1 mg/ml) or PBS (7 μl) was administered locally to the injury site immediately after the trauma. Functional recovery or sensory functions was assessed weekly until week 7, and the results were compared between the sham and the treatment groups.

In the previous two-photon imaging studies, we have shown the effectiveness of recombinant HB-GAM administrated directly to the trauma site in the stimulation of sensory axon outgrowth in a mouse model of dorsolateral transection of the spinal cord (24). To follow the physiological changes occurring both in sensory and motor systems immediately and during the following weeks after the HB-GAM infusion, we implemented the cervical lateral hemisection model that results in disruption of motor and sensory spinal cord tracts and unilateral functional deficit detected in front- and hind-paws. Similar to the two-photon imaging studies mentioned above, HB-GAM was administered locally to the trauma lacuna (7 ul of 1 mg/ml) using a nanofil syringe within a few minutes after the trauma was done and the bleeding was stopped. Two separate cohorts of animals were used to follow the plasticity changes occurring in the activation of the sensory cortex during vibrotactile stimulation in *in vivo* imaging and functional regeneration in repeated behavioral testing during 7–9 weeks after the SCI (Figure 1).

**Figure 2:**
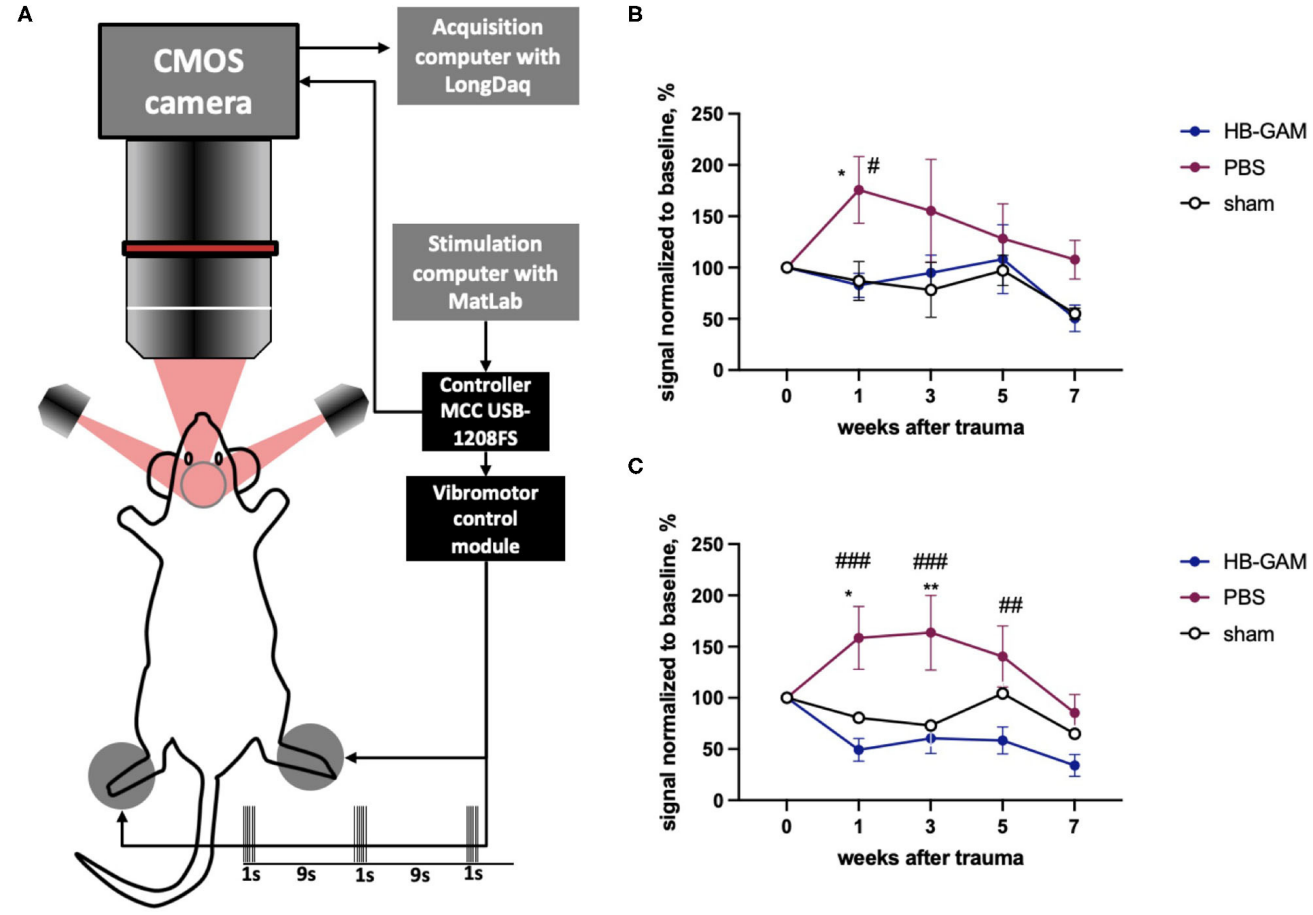
BOLD signals measured from the somatosensory cortex after contra- or ipsilateral vibrotactile hind paw stimulation in mice with the C5 cervical hemisection treated by HB-GAM or PBS locally. **(A)** Schematic representation of the BOLD imaging system. **(B)** Intensity of BOLD signaling normalized to the baseline level measured from the somatosensory cortex during vibrotactile stimulation of the contralateral hind paw. **(C)** Intensity of BOLD signaling normalized to the baseline level measured from the somatosensory cortex during vibrotactile stimulation of the ipsilateral hind paw. ^#^*p* < 0.05, ^##^*p* < 0.01, ^###^*p* < 0.001 vs. the HB-GAM-treated group; *p < 0.05, **p < 0.01 vs. the sham group. *N* = 4–5 in **(B,C)**. Two-way ANOVA followed by Tukey pairwise comparison was used to analyze the group differences at individual time points.

Intrinsic signal optical imaging (ISOI) is similar to functional MRI (fMRI) in that it reveals activity-dependent changes in the nervous system using BOLD imaging (43). In ISOI, signals are obtained by illuminating the brain through an optical window and capturing the reflection using a camera (44). To image the somatosensory cortex on both hemispheres we implemented chronic cleared skull preparation that was transparent enough to map major cortical blood vessels with green reflected light (filter 546BP30) and to follow intrinsic signal using red reflected light (filter 630BP30) for 7 weeks after implantation. Two vibrating motors fixed on hind paws delivered repetitive 1 s-long stimulation trains with 9 s intervals (Figure 2A) and generated a clear 3-phases hemodynamic response in the somatosensory cortex. We assessed the first phase, the so-called “initial deep,” that reflects the decreased amount of oxy-hemoglobin due to oxygen consumption by the nearest actively firing neurons. ISOI signals were assessed from week 1 until week 7 after the surgery in the sham-operated mice and in the mice that had undergone the C5 cervical hemisection and treated with HB-GAM or the vehicle (PBS) only. In general, the signal intensity decreases over time in the sham-operated animals after the surgery in both contralateral and ipsilateral stimulation of the hind paw due to continuous transparency loss of this preparation (Figures 2B,C). Unexpectedly, the ISOI intensity was clearly enhanced after the SCI in the control mice injected with PBS after the SCI. Upon the contralateral vibrotactile stimulation of the hind paw, the BOLD signal was significantly increased at week 1 (Figure 2B) and in the ipsilateral hind paw stimulation at the weeks 1 to 5 in the PBS-treated mice (Figure 2C) compared to the sham-operated mice or the HB-GAM-treated mice. In the HB-GAM-treated mice, the ISOI signaling followed the pattern seen in the sham-operated mice in both contralateral and ipsilateral stimulation from week 1 until the end of the experiment at week 7 without any significant difference compared to the sham-operated animals at any time point (Figures 2B,C). We conclude that HB-GAM is able to block the enhanced neuronal excitability in the somatosensory cortex due to the C5 cervical hemisection.

**Figure 3:**
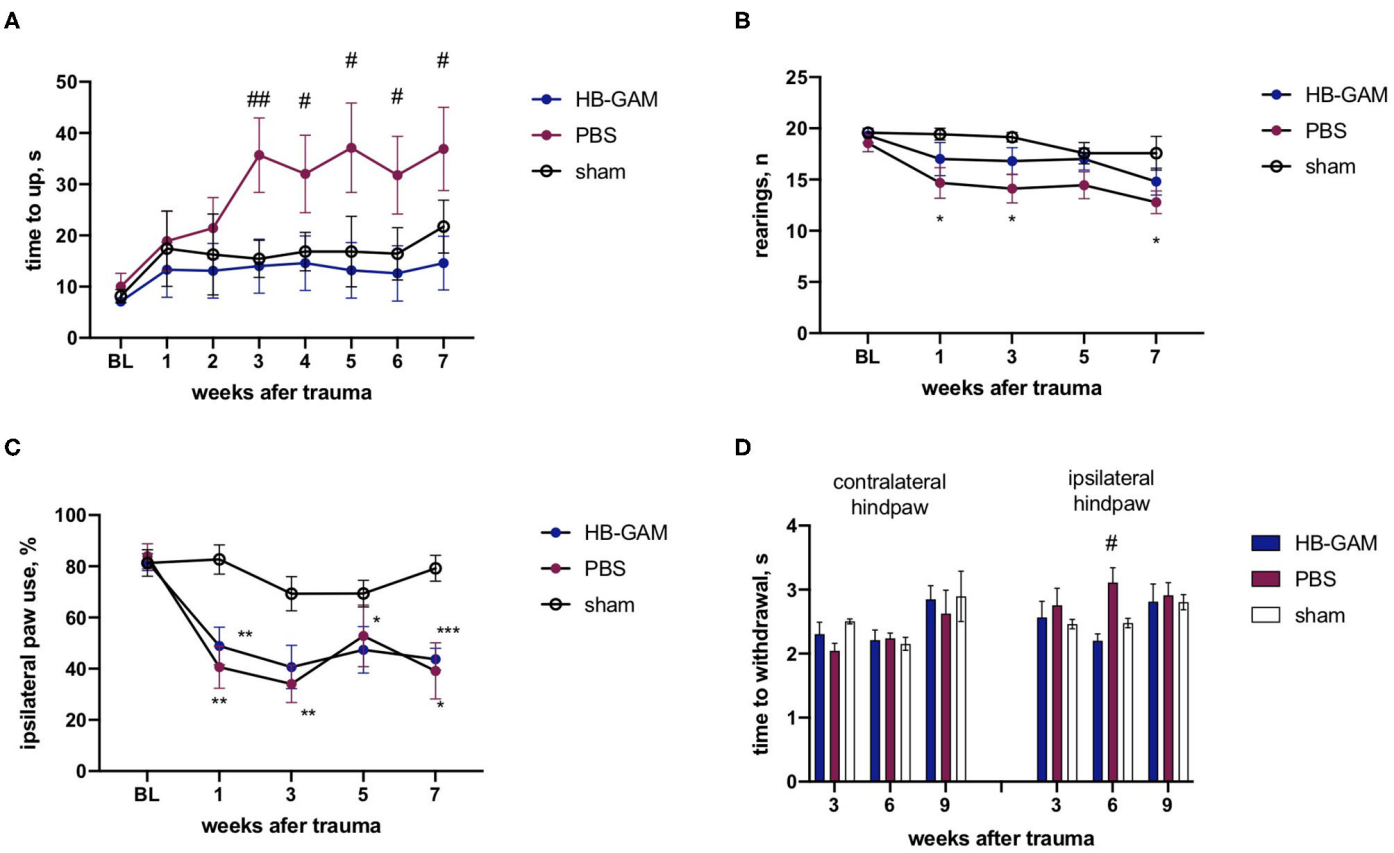
Functional assessment of mice with the C5 cervical hemisection treated by HB-GAM or PBS locally. **(A)** Latency to climb up to the top of the vertical screen measured at different time points after the SCI. **(B)** Number of rearings with front paws used for bodyweight support against the wall performed in the cylinder test. **(C)** Percentage of ipsilateral front paw use for bodyweight support against the wall performed in the cylinder test. **(D)** Time to withdraw for the contralateral and the ipsilateral hind paw measured in the von Frey test. ^#^*p* < 0.05, ^##^*p* < 0.01 vs. HBGAM-treated group; *p < 0.05, **p < 0.01, ***p < 0.001 vs. the sham group. *N* = 6–10 in **(A–D)**. **(A,B)** Kruskal-Wallis test with uncorrected Dunn's pairwise comparison was used to test the group differences at individual time points. **(C,D)** Two-way ANOVA followed by pairwise comparison with Tukey test was used to analyze the group differences at individual time points.

Similar to the ISOI experiments, local injections of HB-GAM into the trauma site were also used to test the possible effects of the compound on motor and sensory-motor behavior. After the SCI the group injected with PBS demonstrated growing deficits in the vertical climbing test requiring coordination of the limb movements. There was a significant group difference between the PBS and the HB-GAM treated animals in latency to climb up to the top of the vertical screen starting from week 3 after the trauma (week 3 *p* = 0.0087, week 4 *p* = 0.0244, week 5 *p* = 0.0217, week 6 *p* = 0.0203, week 7 *p* = 0.0406, Kruskal-Wallis test with uncorrected Dunn's pairwise comparison). In contrast, the HB-GAM-treated animals performed like the sham-operated animals during the whole testing period (Figure 3A). To understand whether the climbing speed depends on the ipsilateral paw use we visually inspected the ability of front paws to grasp the metal bars of the screen each time before the assessment of vertical climbing. There were just few animals with hyperflexion in the ipsilateral paw in both the PBS- and the HB-GAM treated groups (data not shown) while some of them were able to climb on the screen for a short time. Those animals with hyperflexion were not excluded from the analysis.

The asymmetry of the front paw use was assessed with the cylinder test. A significantly lower number of rearings was observed in the PBS-treated group compared to the sham-operated animals during the cylinder test at week 1 (*p* = 0.0106), week 3 (*p* = 0.0163), and the week 7 (*p* = 0.0318, Kruskal-Wallis test with uncorrected Dunn's pairwise comparison), while the HB-GAM treatment partially restored the vertical activity demonstrating the absence of a significant difference vs. the sham animals (Figure 3B). The cervical hemisection resulted in a significant decrease of the ipsilateral paw use in the cylinder test when compared to the sham-operated animals but the HB-GAM treatment did not cause a significant difference compared to the PBS group (Figure 3C).

The attempt to assess allodynia with the von Frey test did not provide conclusive results and demonstrated a big variability between the tested time points: the PBS group demonstrated a tendency to decrease the latency in the contralateral paw withdrawal in comparison with the sham group at the week 3 (*p* = 0.0522, 2-way ANOVA with Turkey *post-hoc* pairwise comparison) which could be interpreted as a sign of allodynia, whereas an increased latency to withdrawal for ipsilateral paw in the PBS group in comparison with the HB-GAM group was observed at the week 6 (*p* = 0.0442, 2-way ANOVA with Turkey *post-hoc* pairwise comparison). Overall, the HB-GAM-treated animals were not different from the sham mice in the von Frey test (Figure 3D).

### Intrathecal Injections of HB-GAM

**Figure 4:**
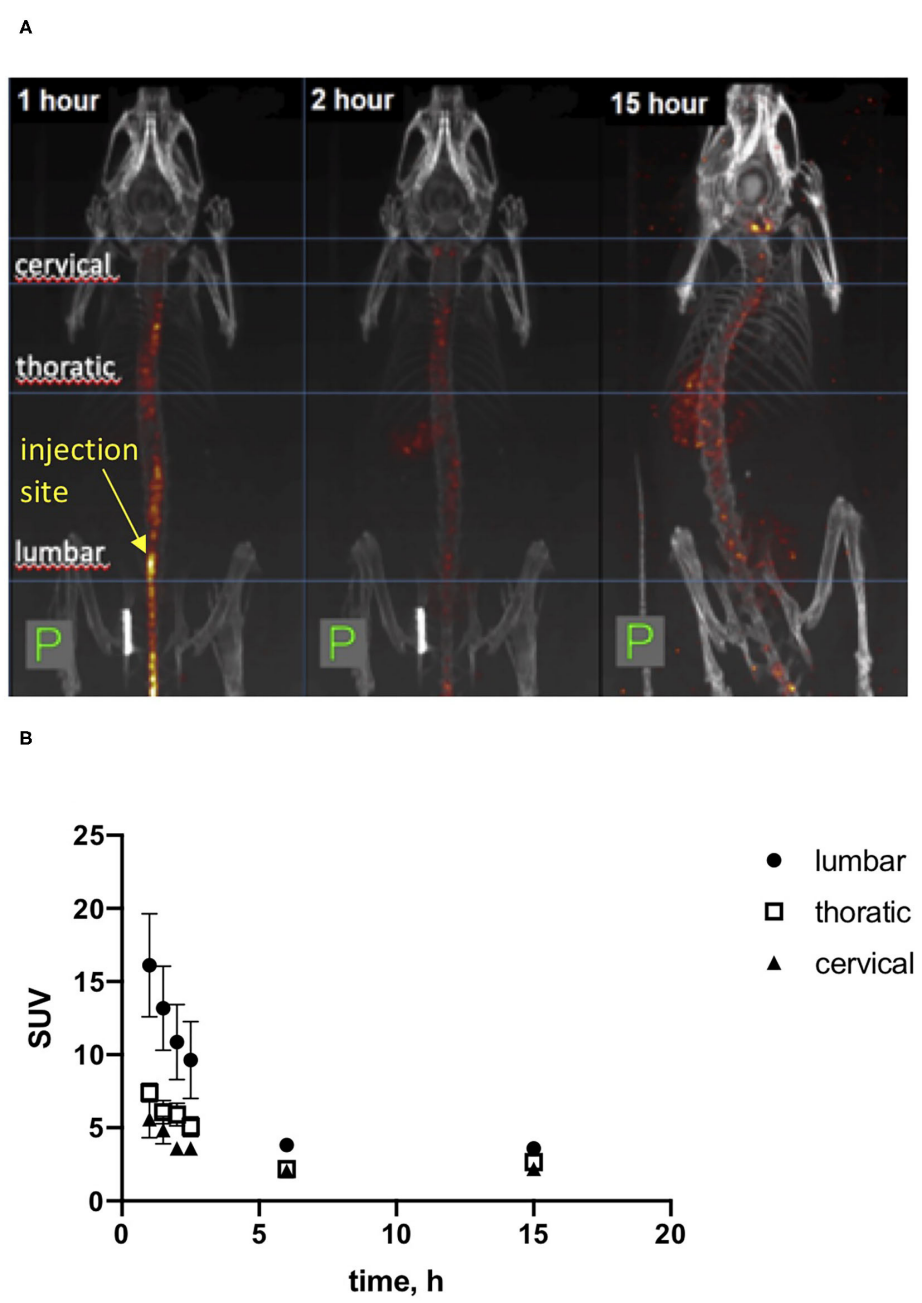
SPECT-CT *in vivo* imaging of [I^123^]HB-GAM distribution through the spinal cord after intrathecal delivery. **(A)** Representative images of mice after intrathecal injection of iodinated HB-GAM. SPECT signal is shown in heat-colored areas. CT is shown in gray. Images were taken after 1, 2, and 15 h after injection of the test compound. **(B)** Standard uptake value for the labeled HB-GAM on several areas of the spinal cord. The first 4 time points are based on two experiments and the later time points on one experiment.

We used initially local injections of recombinant HB-GAM into the trauma site as in our previous studies (24) and the experiments outlined above. To find a delivery route that would be convenient in repeated injections of HB-GAM and could be clinically viable, we tested whether the iodine 123-labeled HB-GAM is able to distribute along the spinal cord and reach the cervical area after intrathecal injection into the lumbar area. SPECT-CT was used to follow HB-GAM distribution. The observed radioactive signal was localized mainly in the spinal canal while γ-signal was also detected from the thyroid, liver, kidney, and urinary bladder at later time points (Figure 4A). The labeled HB-GAM was rapidly taken up by the spinal cord at the place of injection and redistributed to thoracic and cervical regions very fast before the first time point was acquired (around 30 min). The highest uptake value was detected at the first time point and then decreased with time until reaching a plateau after around 2–3 h, which lasted at least 15 h after injection (Figure 4A). Consistently, the highest uptake values were determined in the lumbar area, followed by the thoracic and cervical areas at early points, but the values tended to equalize with time (Figure 4B).

**Figure 5:**
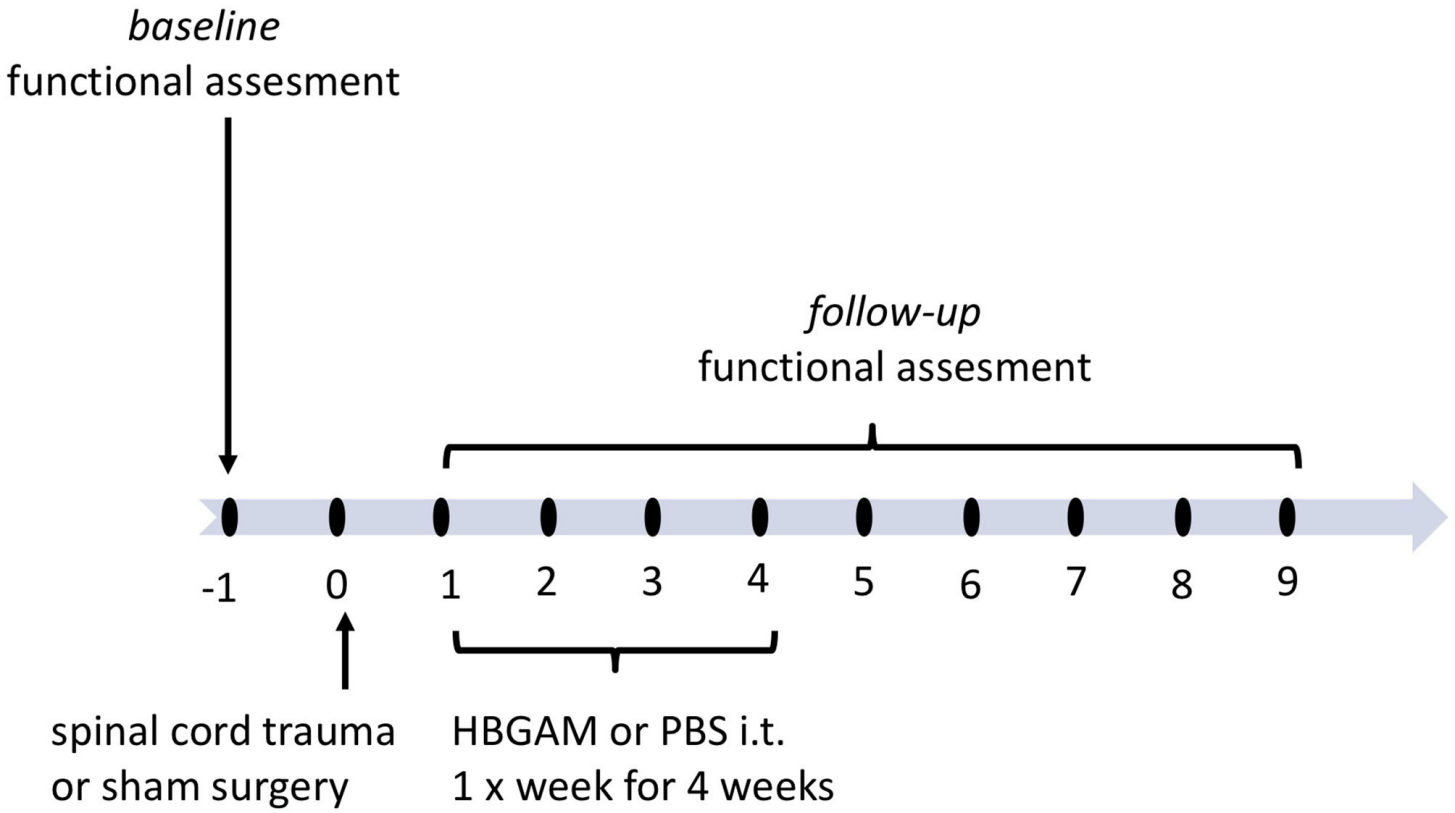
The schedule of treatment and functional assessment in the studies with intrathecally delivered HB-GAM or PBS in hemisection and hemicontusion spinal cord trauma. Animals with spinal cord trauma were equally distributed to treatment groups based on their behavioral results measured at week one after the surgery. HBGAM (7 μl, 1 mg/ml) or PBS (7 μl) was administered intrathecally once per week for four weeks starting from week one. The sham-operated animals received PBS injections in the same manner as the HB-GAM-treated animals after the injury. Group differences were analyzed for each individual timepoint. Additionally, functional recovery was assessed repeatedly until week 7 or 9 and compared to the level performed at week 1 when treatment was just started.

Since our delivery protocol using intrathecal HB-GAM injection resulted in the distribution of the labeled protein to the cervical area, we started experiments to test the effectiveness of HB-GAM administrated repeatedly through intrathecal injections in the mouse model C5 hemisection. As a difference from the local delivery (Figure 1), the treatment was started during the subchronic period that is more translatable to the clinical situation when there is no possibility or intention to start invasive treatment within the first few hours after the SCI. One week after the spinal cord trauma animals were slightly anesthetized by isoflurane and 7 μl of 1 mg/ml HBGAM or the vehicle (PBS) was injected by intrathecal needle prick through the skin. Injections were repeated once per week for 4 weeks (Figure 5).

**Figure 6:**
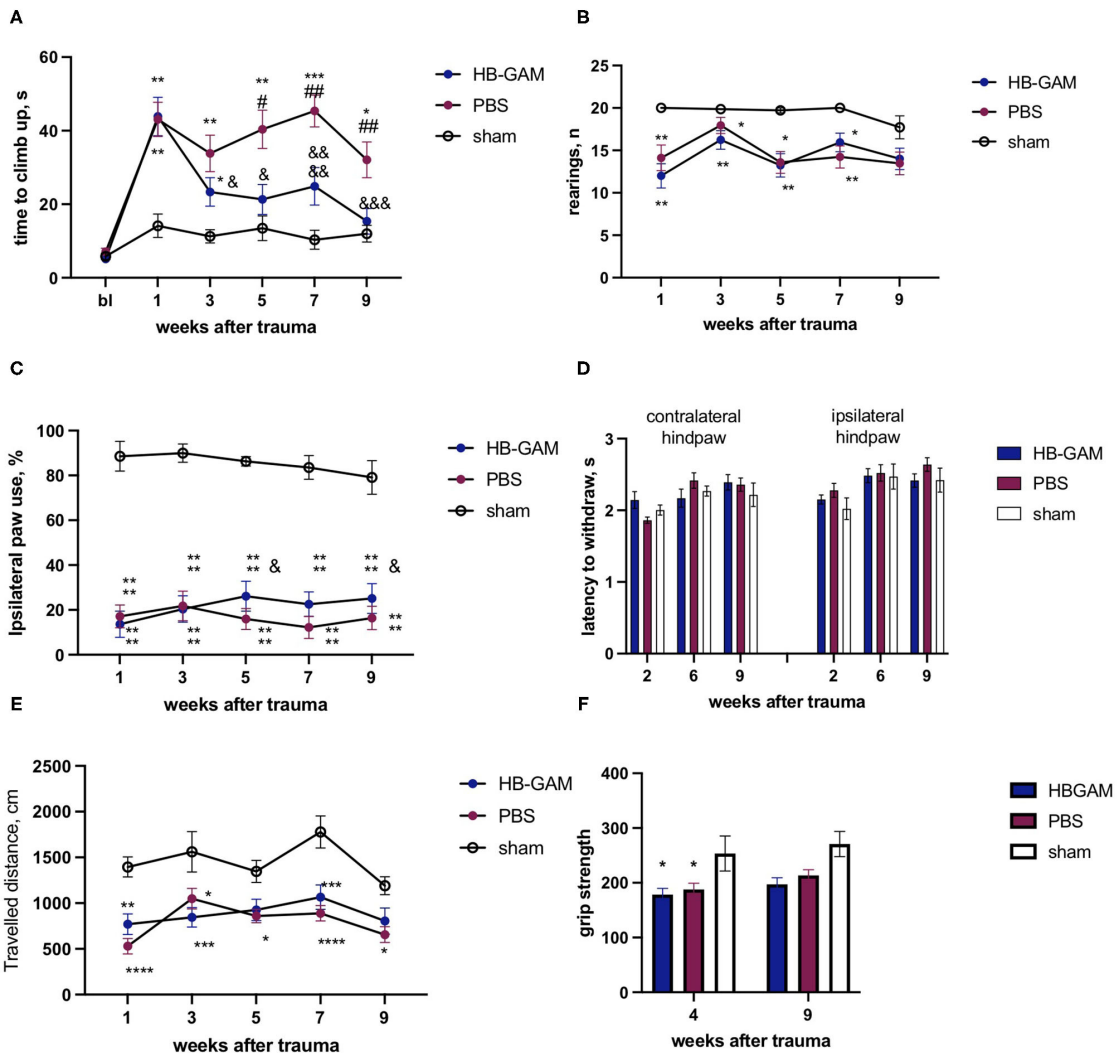
Functional assessment of mice with the C5 cervical hemisection treated by intrathecally delivered HB-GAM or PBS. **(A)** Latency to climb up to the top of the vertical screen measured at different time points after the SCI. **(B)** Number of rearings with front paws used for bodyweight support against the wall performed in the cylinder test. **(C)** Percentage of ipsilateral front paw use for bodyweight support against the wall performed in the cylinder test. **(D)** Time to withdraw for the contralateral and the ipsilateral hind paw measured in the von Frey test. **(E)** Distance traveled in an open field within 15 min testing session measured at different time points after the SCI. **(F)** Grip strength assessed for the ipsilateral front paw at weeks 4 and 9 after the trauma. ^&^*p* < 0.05, ^&&&^*p* < 0.001, ^&&&&^*p* < 0.0001 vs. the week 1 for the same group. ^##^*p* < 0.01 vs. the HBGAM-treated group; *p < 0.05, **p < 0.01, ***p < 0.001, ****p < 0.0001 vs. the sham group. *N* = 7–17 in **(A–F)**. **(A,B)** Kruskal-Wallis test with uncorrected Dunn's pairwise comparison was used to test the group differences at the individual time points. Friedman test with Dunn's *post-hoc* pairwise comparison was used to compare the individual timepoints with the level measured at week 1 within the same group. **(C–F)** Two-way ANOVA followed by pairwise comparison with Tukey test and Dunnett test were used to analyze the group differences at the individual time points and to compare the individual timepoints with the level measured at the week 1 respectively.

In the experimental schedule using intrathecal administration, the mice that had undergone hemisection displayed a clearly increased time in the climbing test (Figure 6A). The HB-GAM-treated mice clearly displayed recovery until the end of the experiment at the week 9: there were significant within-group differences during the weeks 5 (*p* = 0.0354), 7 (*p* = 0.0408), and 9 (*p* = 0.005, Friedman test with Dunn's pairwise comparison) vs. the pre-treatment value at the week 1, and there was no group difference vs. the sham animals at the weeks 5 and 9. In contrast to the HB-GAM-treated animals, no recovery was observed in the PBS-treated mice: starting from week 1 the PBS animals constantly demonstrated a significantly longer climbing time than the sham animals. Moreover, the climbing time for the PBS group vs. the HB-GAM group was significantly longer at weeks 5 (*p* = 0.0136), 7 (*p* = 0.0041), and 9 (*p* = 0.0046, Kruskal-Wallis test with uncorrected Dunn's pairwise comparison) (Figure 6A). Similar to the experiment with local HB-GAM administration, we visually inspected and scored the ability of the animals to grasp the bar of the vertical screen before each vertical screen test. While there were some animals in both groups having hyperflexed ipsilateral front paw and limited abilities to use the paw, no difference could be observed between the PBS and the HB-GAM group.

Spinal cord trauma resulted in a significant decrease of rearings performed in the cylinder test compared to the sham-operated animals; the animals treated with HB-GAM or PBS did not display any significant difference (Figure 6B). In the percentage of ipsilateral paw use for rearing support against the wall, HB-GAM induced some improvement of ipsilateral paw use at the weeks 5 (*p* = 0.0282) and 9 (*p* = 0.027, two-way ANOVA with Dunnett pairwise comparison) compared to the level at the week 1 (Figure 6C). The percentage of ipsilateral paw use in the PBS group use stayed constantly low during the whole experimental period (Figure 6C).

Similar to the experiment with local treatment, only some minor allodynia signs were detected in the PBS group during the first weeks after the SCI: the von Frey test revealed a trend for decreased latency in contralateral paw withdrawal at week 2 in the PBS group vs. the HB-GAM group (*p* = 0.09, Figure 6D), while no any other differences were detected at later timepoints for contralateral or ipsilateral hind paws.

Spontaneous locomotor activity was tested in the open field test to understand whether the improved climbing in the HB-GAM group was associated with an increase in general arousal and exploratory behavior. The spinal cord trauma significantly decreased the distance traveled in the open field for both the HB-GAM and the PBS groups compared to the sham group, without any visible improvement during the whole experimental period. Locomotor activity was quite similar in the PBS and the HB-GAM group at all time points tested (Figure 6E).

There was no trauma or treatment effect of the grip strength in the contralateral front paw, while the strength of the ipsilateral paw was reduced in the SCI animals compared to the sham group without any significant difference between the groups treated with HB-GAM or PBS (Figure 6F).

Starting from week 1 after the spinal cord trauma all animals progressively increased body weight without group differences at any tested time point (data not shown). as was also found in the grip strength tests (Figure 6F).

**Figure 7:**
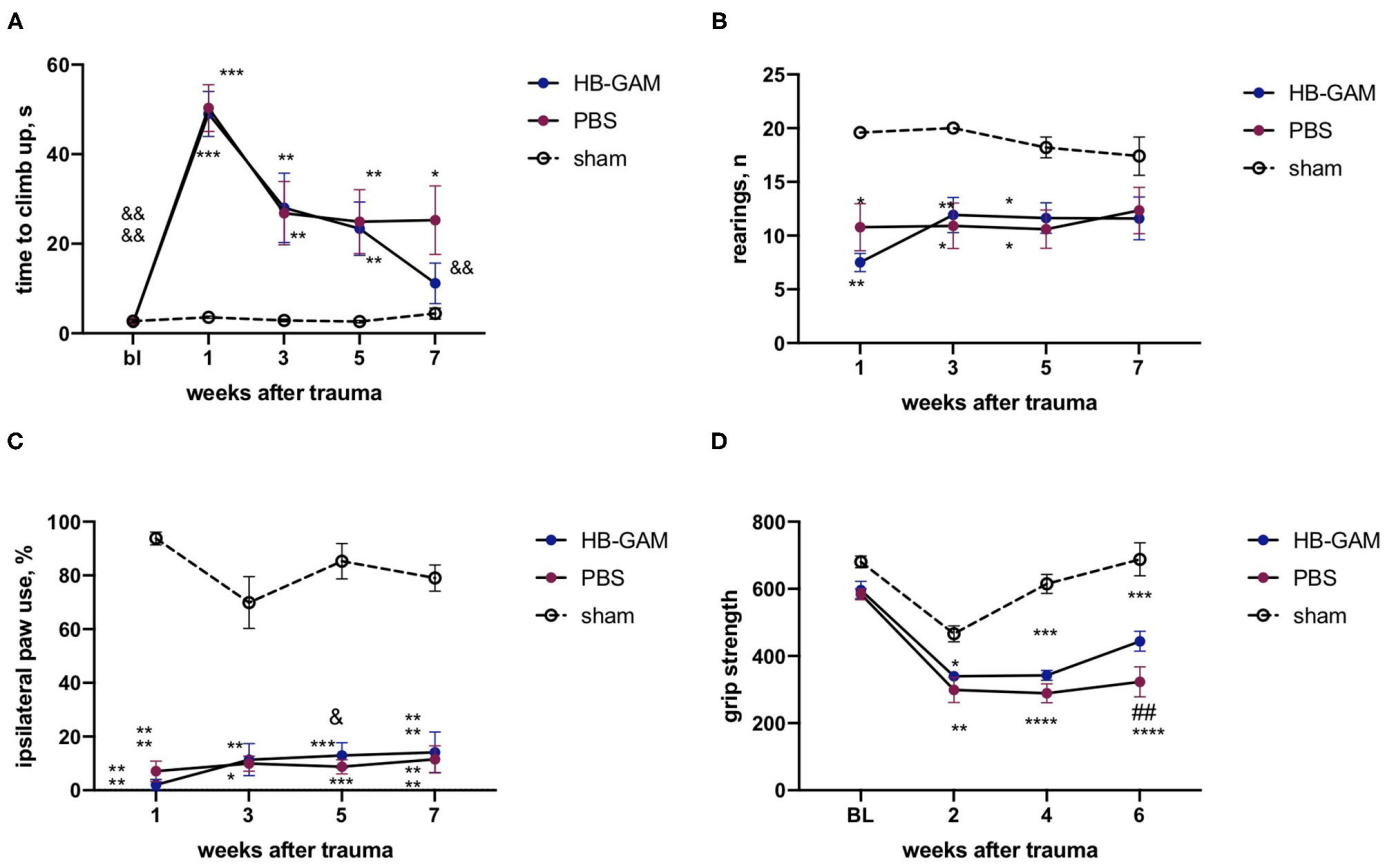
Functional assessment of mice with the C5 cervical hemicontusion treated by intrathecally delivered HB-GAM or PBS. **(A)** Latency to climb up to the top of the vertical screen measured at different time points after the SCI. **(B)** Number of rearings with front paws used for bodyweight support against the wall performed in the cylinder test. **(C)** Percentage of ipsilateral front paw use for bodyweight support against the wall performed in the cylinder test. **(D)** Front paws grip strength measured at different time points after the SCI. ^&^*p* < 0.05, ^&&^*p* < 0.01, ^&&&&^*p* < 0.0001 vs. the week 1 for the same group; ^##^p < 0.01 vs. the HB-GAM-treated group; *p < 0.05, **p < 0.01, ***p < 0.001, ****p < 0.0001 vs. the sham group. *N* = 5–11 in **(A–D)**. **(A,B)** Kruskal-Wallis test with uncorrected Dunn's pairwise comparison was used to test the group differences at the individual time points. Friedman test with Dunn's *post-hoc* pairwise comparison was used to compare the individual timepoints with the level measured at week 1 within the same group. **(C,D)** Two-way ANOVA followed by pairwise comparison with Tukey test and Dunnett test were used to analyze the group differences at the individual time points and to compare the individual timepoints with the level measured at the week 1 respectively.

Since the effects on motor behavior may be revealed in a different manner in different trauma models, we established a hemicontusion model at the C5 cervical level and used this model in addition to the C5 hemisection model (see above). The severity of trauma was controlled by the automatically assessed impact force that was similar in both groups with spinal cord contusion (PBS group 88.5+/−3.9 K Dynes, HB-GAM group 87.9+/−2.4 K dynes). As in the hemisection model, hemicontusion resulted in a significant increase in the climbing time that was spontaneously reduced both in the PBS-treated and in the HB-GAM-treated groups, while the climbing time reduction reached statistical significance only in the HB-GAM group at the week 7 (*p* = 0.0025, Friedman test with Dunn's pairwise comparison) in comparison with the climbing speed at week 1 after the SCI (Figure 7A). Similar to the hemisection trauma, there was no difference in grasping abilities measured just before the climbing test between the PBS and the HB-GAM groups (data not shown).

No detectable treatment effect in the cylinder test was observed in vertical activity (number of rearings, Figure 7B) or the use of the injured paw (Figure 7C); both the PBS and the HB-GAM group demonstrated significant impairment of cylinder test performance in comparison with the sham animals. The HB-GAM group displayed some increase in ipsilateral paw use at week 5 (*p* = 0.038, 2-way ANOVA with Dunnett pairwise comparison) (Figure 7C) in comparison with the pretreatment level at week 1.

Hemicontusion caused a clear decrease in the front paw strength compared to the sham-operated animals. To be able to measure the grip strength at an earlier timepoint after the SCI in comparison with the data obtained in the hemisection model, we have implemented another approach using an automated grip strength meter assessing the cumulative grip strength for both front paws simultaneously. The grip strength values of the HB-GAM group slowly recovered over the experimental period, reaching a significant improvement in comparison with the PBS group at week 6 (*p* = 0.005, 2-way ANOVA with Turkey *post-hoc* pairwise comparison) (Figure 7D). As in the hemisection model (Figure 6E), the overall spontaneous activity that depends on both the mental and physical status of the animals was similar in the groups with hemicontusion (not shown).

## Discussion

Heparin-binding growth-associated molecule (HB-GAM) was initially isolated by screening for factors that enhance neurite outgrowth in central neurons (22). Molecular cloning confirmed that HB-GAM (pleiotrophin) is a novel neurite outgrowth-promoting factor that is highly expressed in the developing brain (up to 10 μg/g wet tissue weight) and binds to heparin (25, 28). HB-GAM is a secretory protein that lines developing fiber tracts in the rat brain but is downregulated in the adult CNS (25, 45).

Properties of HB-GAM, such as the neurite outgrowth-enhancing effect and the expression that peaks in the perinatal rodent brain corresponding to the critical period of development with heightened plasticity, suggest a role for HB-GAM in plasticity regulation of neuronal connections. We have, therefore, more recently started studies on the role of HB-GAM in plasticity and regeneration after SCI. In SCI, chondroitin sulfate proteoglycans of the glial scar are known to inhibit plasticity and regeneration (2) and our focus has been on chondroitin sulfate chains that were previously shown to bind HB-GAM at nanomolar K_D_ values (23). Our *in vitro* studies have shown that HB-GAM is able to reverse the inhibition of neurite outgrowth by chondroitin sulfates; in these assays chondroitin sulfates even display a positive role in neurite outgrowth *via* binding of HB-GAM and presenting it to neurons (24, 46). Furthermore, our *in vivo* studies have shown that after a single injection into the site of prick-injury area HB-GAM accumulates to the area of astrocytes forming the inhibitory glial scar and stays there detectable until at least 20 days after the injection. Two-photon microscopy in live animals has shown that the injected HB-GAM enhances axon growth through the injury site starting 2 weeks and continuing at least until 4 weeks after a section injury of the spinal cord (24). However, possible functional outcomes of HB-GAM administration in SCI models have not been so far studied.

The choice of the SCI model is a traditional question for discussion in preclinical studies: the section model results in an accurate and reproducible lesion that is perfect for studies in laboratory conditions, while the contusion model reproduces better the pathological mechanisms of the human SCI (31, 32). Moreover, drastic temporal and spatial differences have been demonstrated in GSPG deposition and retraction of corticospinal tract axons in rat models of section and contusion SCI (16). A higher level of GSPG accumulation and gliosis in much more extended tissue area around trauma site in contused vs. sectioned spinal cord could underly substantial differences in treatment efficacy. Our previous two-photon imaging study demonstrated the outgrowth of sensory axons through the lesion site after local HB-GAM treatment in the dorso-lateral transection model (24). As in our two-photon imaging studies, we used in the current study incomplete SCI and studied the HB-GAM effects in lateral hemisection and lateral hemicontusion mouse models that are known to produce measurable asymmetric behavioral effects. Therefore, in the current study, we first used the cervical (C5) lateral hemisection model to test the restorative potential of recombinant HB-GAM in functional regeneration of the posttraumatic spinal cord and after that implemented the cervical hemicontusion model to validate the obtained results.

To study sensory signaling after hemisection at the C5 level, we have measured BOLD (blood oxygenation level-dependent) signaling in the somatosensory cortex induced by vibrotactile stimulation of the hind paw. While the cervical lateral SCI results in a change of motor and sensory function both in front and hind paws, stimulations of hind paws allow assessing of the functioning of ascending pathways and their representation in the somatosensory cortex with minimum interactions with possible pathological processes in the peripheral or central nervous system on the level of trauma. We have applied the technique designated ISOI that is in principle similar to BOLD fMRI. Interestingly, the ISOI signals due to vibrotactile hind paw stimulation are clearly enhanced after the spinal cord hemisection on the area of the hind paw representation in the somatosensory cortex (Figures 2B,C). Exaggerated responsiveness of spinal neurons has been previously demonstrated below the trauma site using BOLD fMRI (47). Furthermore, hyperexcitability in the somatosensory cortex has been shown immediately after spinal cord hemisection (48) and unilateral spinal injuries have been shown to result in bilateral hyperexcitability in thalamic neurons (49). These findings appear similar to our findings using BOLD imaging in the somatosensory cortex after hind paw stimulation.

Heparin-binding growth-associated molecule (HB-GAM) injected into the trauma site strongly reduces the ISOI signals in the somatosensory cortex. At all time points measured (from week 1 until week 7) the ISOI signals in the HB-GAM-treated mice are at the level seen in the sham-operated mice (Figures 2B,C). Loss of descending inhibition probably contributes to exaggerated responsiveness of spinal neurons (50) and is likely replaced by inhibitory tone introduced by HB-GAM injected to the trauma area or given by intrathecal injections. HB-GAM has been previously shown to enhance GABA_A_-mediated inhibition (51). Furthermore, the GABA_A_ receptor has been shown to enhance neurite outgrowth (52), which might aid in the growth of neurites through the trauma site in addition to the chondroitin sulfate/HB-GAM mechanism studied previously (24).

Unilateral lesions of the spinal cord cause problems in skilled locomotion (53). This was also observed in the current study in which the most constant effect on locomotion in different tests after unilateral lesions was seen in climbing along the vertical ladder requiring coordination of the limb movements. Performance in the climbing test was clearly enhanced after hemisection by HB-GAM injected locally into the trauma site (Figure 3A) or given intrathecally (Figure 6A), and some improvement was also seen in the hemicontusion model (Figure 7A). The similarity in the profile of behavioral recovery after a single HB-GAM bolus administered immediately after the trauma and repeated HB-GAM treatment started 1 week after the trauma exposure in hemisection SCI suggests that the underlying mechanisms of the observed effect more likely relate to the promotion of regenerative activity than to the regulation of acute immune response developed within the first few days after SCI. Nevertheless, the discovered abilities of HB-GAM (pleiotrophin) to modulate the microglia (54) and leukocytes (55) could have some impact on inflammatory response after SCI and are worth to be explored more in the following studies. Moreover, additional studies including thoughtful histological analysis are required to understand the underlying anatomical mechanism of the observed functional improvements and to differentiate the therapeutic potential of HB-GAM treatment in hemicontusion and hemisection types of SCI.

As a difference from the climbing test, HB-GAM administration did not affect spontaneous locomotor activity that depends on both physical and mental status of the animals and was somewhat reduced in both the PBS-treated and the HB-GAM-treated SCI mice compared to the sham-operated animals. The HB-GAM effect in demanding tasks such as the climbing test or the cylinder test cannot be therefore simply explained by enhanced general activity, motivation, or arousal of the mice but rather suggests a specific effect on neuronal circuits required in coordinated motor functions.

Locomotion in a complex environment requires integrated spatiotemporal information that is processed in the somatosensory cortex (56). Interestingly, HB-GAM normalized excitability in the somatosensory cortex upon vibrotactile stimulation and performance in the climbing tests to the level seen in the sham-operated animals. It appears reasonable that the abnormal hyperexcitability seen in the somatosensory cortex upon the hind paw stimulation is linked to problems in skilled movement. Hyperexcitability in the somatosensory cortex may perturb information processing required in demanding locomotion tasks. Further studies on the effects of cortical hyperexcitability on motor functions are currently warranted.

Hyperalgesia is often observed in spinal cord lesions (33, 35) and hyperexcitability observed in the current study could be linked to the development of hyperalgesia. One of the most popular behavioral protocols for the assessment of tactile sensitivity and hyperalgesia is the von Frey test, which can be used in both humans and animals. While humans can describe the modality of their sensation verbally, the latency to the paw withdrawal is used as the marker of an unpleasant painful sensation in animals. In the animals, with spinal cord trauma, the withdrawal reaction can be affected by impaired motor abilities that could lead to misinterpretation of the results and wrong conclusions. In our experimental models, we only observed some signs of aberrant pain sensation that were detected using the von Frey test, which warrants further studies.

It is also noteworthy that factors inducing sprouting of neurites are known to lead to the development of hyperalgesia. For example, overexpression of nerve growth factors is known to cause hyperalgesia (57). HB-GAM was initially isolated as a factor inducing sprouting of neurites, and one could therefore expect that it causes hyperalgesia that should be seen in the von Frey tests. However, we did not observe any signs of hyperalgesia in our experiments where rather high doses of HB-GAM were injected locally or through intrathecal administration. GABAergic signaling is well-known for its analgesic activity, and the property of HB-GAM to enhance GABAergic activity (51) might counteract in development of hyperalgesia. From this viewpoint, HB-GAM might be an ideal neurite outgrowth-enhancing factor in SCI treatment.

A decrease in the grip strength of the forepaws is one parameter to follow healing after cervical injury of the spinal cord and was found in the hemicontusion model but not in the hemisection model in the current study. The comparison of the hemisection and hemicontusion results in the grip strength test is not very accurate due to a technical difference in the method: in the hemisection model the grip strength was assessed separately for ipsi- and contralateral paws, while cumulative grip strength measurement for both paws simultaneously was used in the hemicontusion study. To address this question, we tested a separate batch of animals with cervical hemisection exactly in the same test of cumulative grip strength assessment as in hemicontusion (data not shown). These data confirmed the lack of effect for HB-GAM in the grip strength in the hemisection model and the beneficial effect of HB-GAM treatment in the vertical screen test.

The loss of grasping abilities of the front paws after cervical spinal cord injury is driven mostly by disruption of descending pathways and partially by damage of segmental circuity including motoneurons loss on the level of the trauma (58, 59). Contusion SCI is characterized by a more extended lesion area and secondary damage that could provide a greater impact to segmental circuity impairment in comparison with section SCI. On the other side, the hemisection SCI model is more precise and accurate leaving fewer chances for spared neuronal tissue at least in conditions of laboratory experiment. In the rat study with C5 spinal cord hemisection, Anderson et al. found that animals with complete hemisection demonstrated irreversible loss of grasping function in ipsilateral front paw while the animals with spared corticospinal tract partially restored grip strength within a few weeks (60). Anatomical peculiarities and existence of some amount of spared tissue available for plasticity stimulation by HB-GAM in contusion trauma could provide some explanation for the observed beneficial effect of HB-GAM treatment in hemicontusion SCI and absence of grip strength recovery in mice with hemisection trauma. HB-GAM enhanced recovery of the grip strength compared to the PBS-treated animals in the contusion model (Figure 7D) but recovery of the grip strength did not reach the level of the sham-operated animals as was seen in the tests of skilled locomotion. It is possible that restoring the grip strength ability back to the sham level following hemicontusion would require some combinational therapy that would enhance the plasticity of the spared tissue and recovery of damaged segmental circuity on the level of trauma in addition to HB-GAM-induced plasticity stimulation.

Recently discovered the copy-number variant in the Dock2 gene and altered immunological phenotype of C57Bl6/NHsd (61) raises questions regarding the possible impact of selected animal strain on neurodegenerative progress and recovery following SCI in our study. Considering the important role of inflammatory response in SCI (1, 7) this aspect should be addressed experimentally by including a few mouse strains with striking different genetic backgrounds in the study design in further studies.

It appears likely that the capability of HB-GAM to enhance plasticity and regeneration based on its interactions with chondroitin sulfate proteoglycans in the glial scar (24) contributes to functional recovery from SCI. Furthermore, the HB-GAM effects on sensory signaling observed in the current study may contribute to recovery seen in skilled locomotion requiring integration of afferent and efferent signaling in the somatosensory cortex. It is noteworthy that the HB-GAM-treated animals appear quite similar to the sham-operated animals in that they do not display exaggerated BOLD signaling detected by the ISOI method in the somatosensory cortex after stimulation of the hind paw. We suggest that HB-GAM replaces the lost inhibitory tone that underlies the exaggerated BOLD signaling due to its capability to enhance GABA_A_ergic activity (51) that is required for normal spinal cord functions. Due to its effects on plasticity/regeneration and GABAergic signaling HB-GAM could pave the way for drug development to treat injuries of the spinal cord.

## Data Availability Statement

The raw data supporting the conclusions of this article will be made available by the authors, without undue reservation.

## Ethics Statement

The animal study was reviewed and approved by National Animal Experiment Board of Finland (Eläinkoelautakunta ELLA).

## Author Contributions

NK designed and carried out the animal surgeries and the behavioral experiments and performed the data analysis. DM developed the protocol for BOLD signaling assessment. SS and DM performed optical windows installations, BOLD imaging, and BOLD data analysis. EM performed behavioral experiments and assisted in animal surgeries. AG designed and performed SPECT-CT study of the compound distribution, imaging analysis, and data interpretation. NK, MP, and HR participated in the study design and data interpretation. HR provided funding and study administration. All authors contributed to manuscript preparation.

## Funding

The study was funded by the Wings for Life foundation, the Academy of Finland, and the Sigrid Jusélius foundation. SPECT/CT imaging was performed at the Real-Time Imaging lab, supported by Biocenter Finland and Helsinki Institute of Life Science. Animal experiments were done with the use of facilities and equipment of Mouse Behavioral Phenotype Facility (MBPF), supported by Biocenter Finland and Helsinki Institute of Life Science.

## Conflict of Interest

The authors declare that the research was conducted in the absence of any commercial or financial relationships that could be construed as a potential conflict of interest.

## Publisher's Note

All claims expressed in this article are solely those of the authors and do not necessarily represent those of their affiliated organizations, or those of the publisher, the editors and the reviewers. Any product that may be evaluated in this article, or claim that may be made by its manufacturer, is not guaranteed or endorsed by the publisher.
